# Supplementary figures and images for: Transcription-associated topoisomerase 2α (TOP2A) activity is a major effector of cytotoxicity induced by G-quadruplex ligands
Source: eLife. 2021 Jun 28;10:e65184. doi: 10.7554/eLife.65184 (PMC8279764; doi:10.7554/eLife.65184)

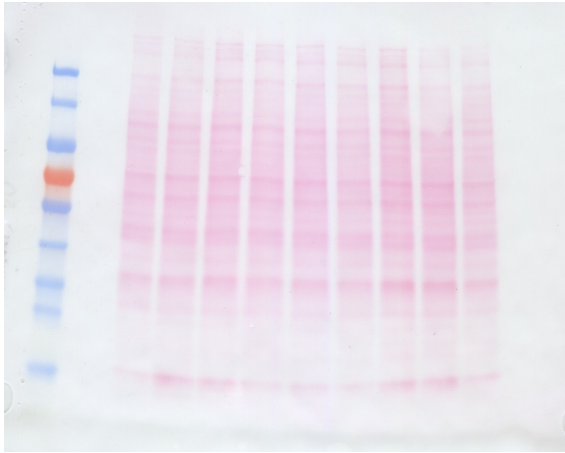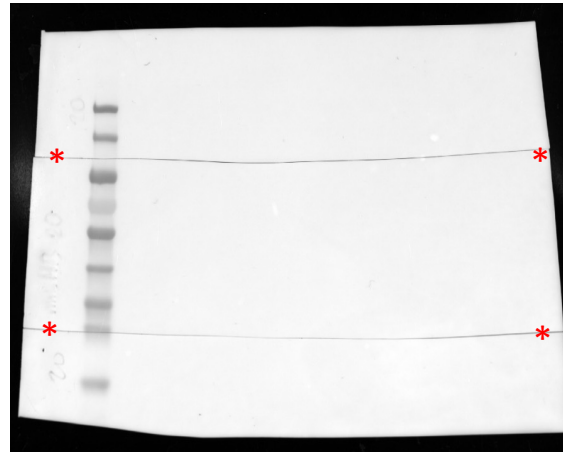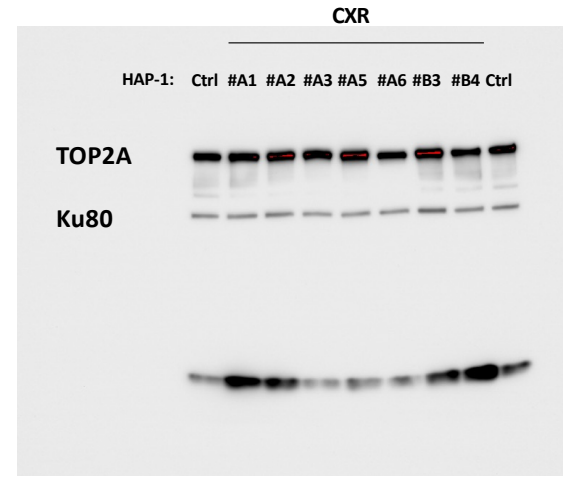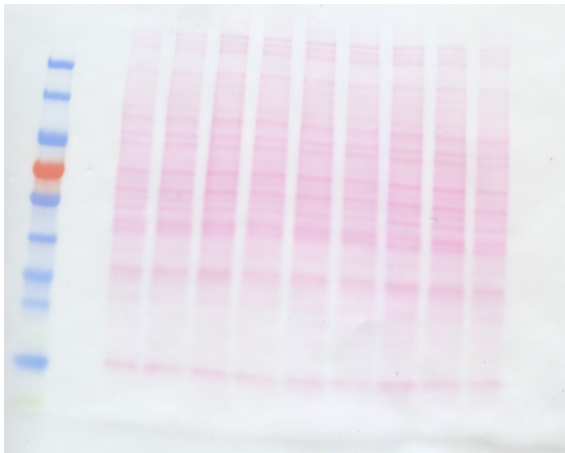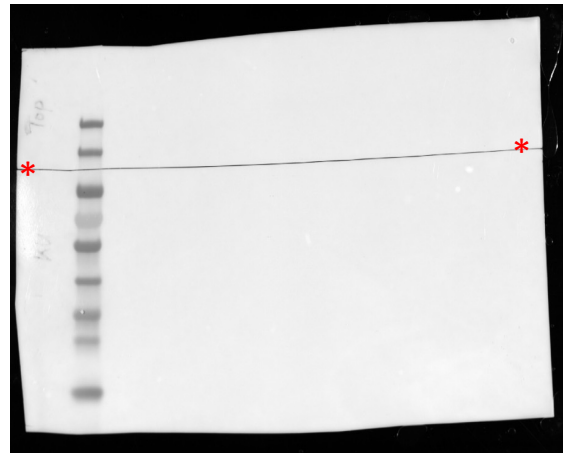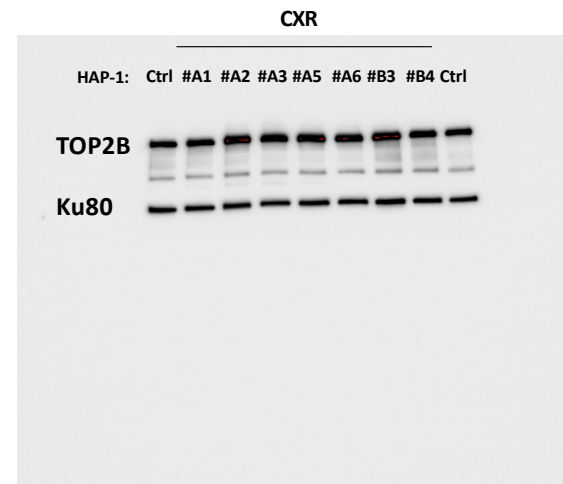

Supplement: Figure 1—source data 1. — Ponceau staining (left panels), membranes (center panels), and hybridization signals (right panels) are shown. Raw images were acquired using the ChemiDoc system (Bio-Rad). Asterisks indicate the edges of cut membranes before hybridization. [file elife-65184-fig1-data1.pdf]

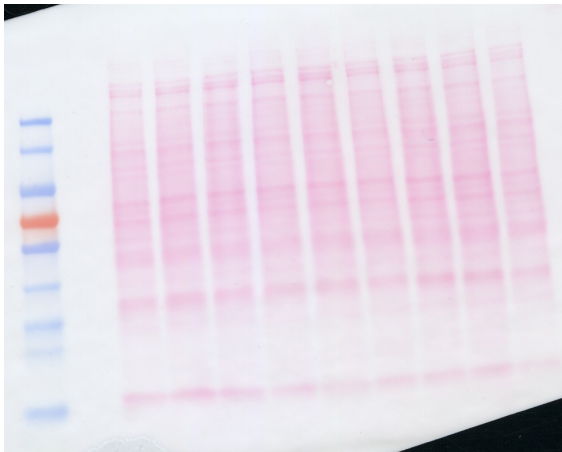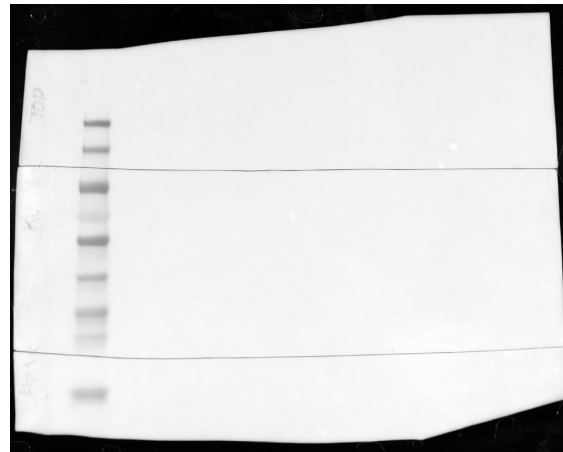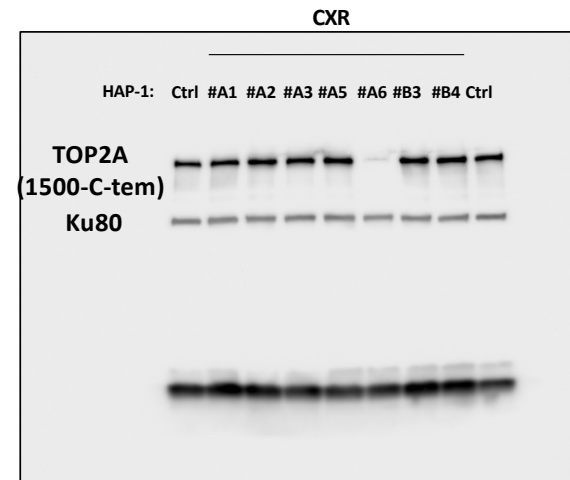

Supplement: Figure 1—figure supplement 1—source data 1. — Ponceau staining (left panel), membrane (center panel), and hybridization signals (right panel) are shown. Raw images were acquired using the ChemiDoc system (Bio-Rad). Asterisks indicate the edges of cut membranes before hybridization. [file elife-65184-fig1-figsupp1-data1.pdf]

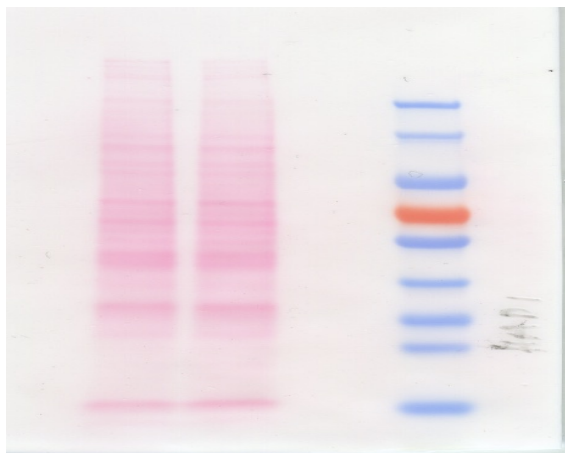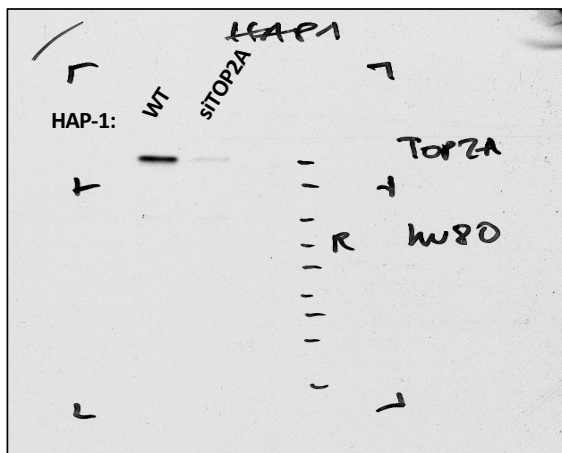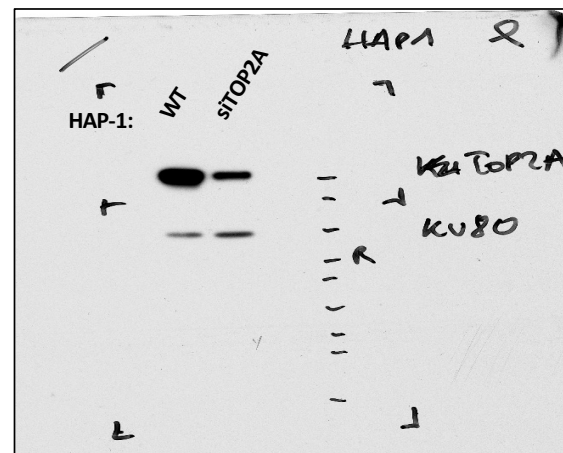

Supplement: Figure 2—source data 1. — Ponceau staining (left panel) and hybridization signals (center and right panel) are shown. Raw images correspond to the scanning of autoradiography films. [file elife-65184-fig2-data1.pdf]

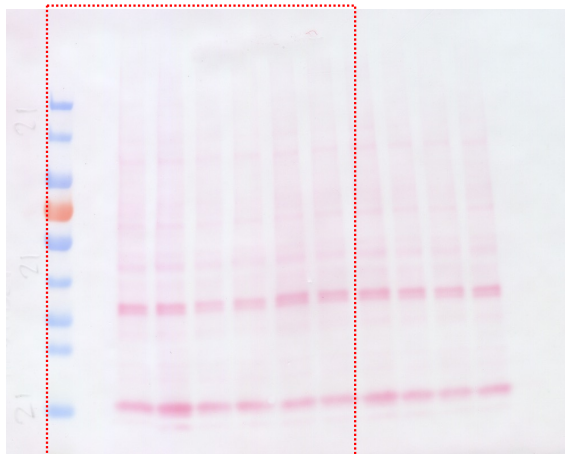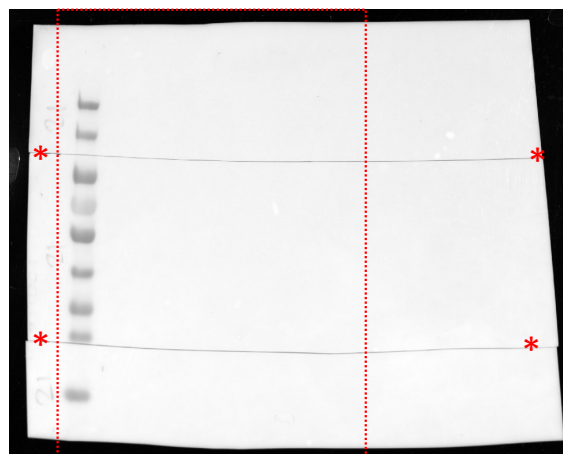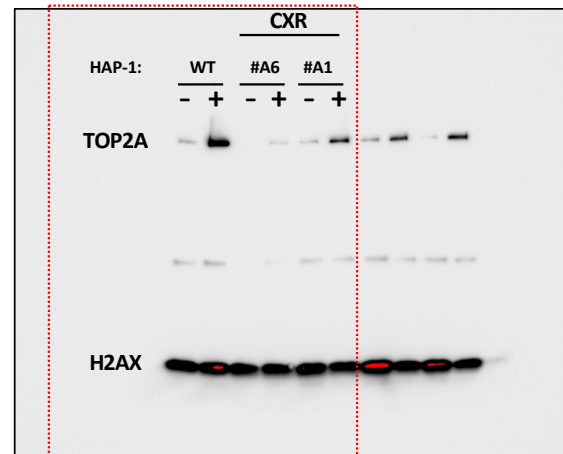

Supplement: Figure 2—figure supplement 1—source data 1. — Ponceau staining (left panel), membrane (center panel), and hybridization signals (right panel) are shown. Raw images were acquired using the ChemiDoc system (Bio-Rad). Asterisks indicate the edges of cut membranes before hybridization. The section of the blot used for the final figure is indicated by the dashed rectangle. [file elife-65184-fig2-figsupp1-data1.pdf]

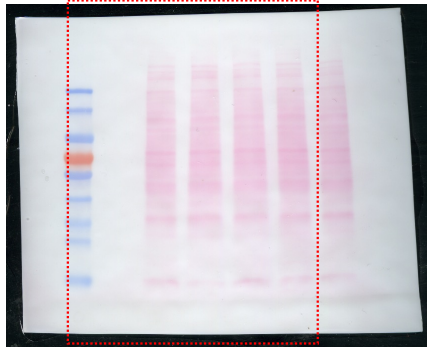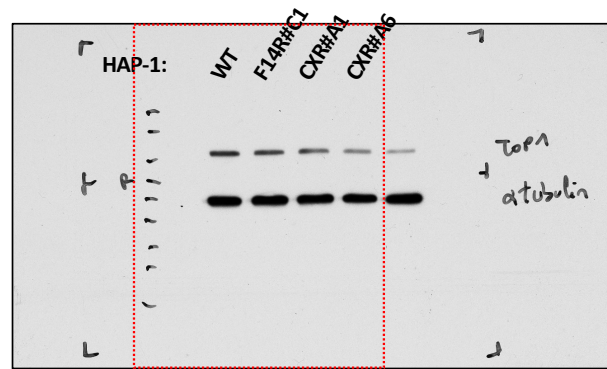

Supplement: Figure 2—figure supplement 2—source data 1. — Ponceau staining (left panel) and hybridization signals (right panel) are shown. Raw images correspond to the scanning of autoradiography films. The section of the blot used for the final figure is indicated by the dashed rectangle. [file elife-65184-fig2-figsupp2-data1.pdf]

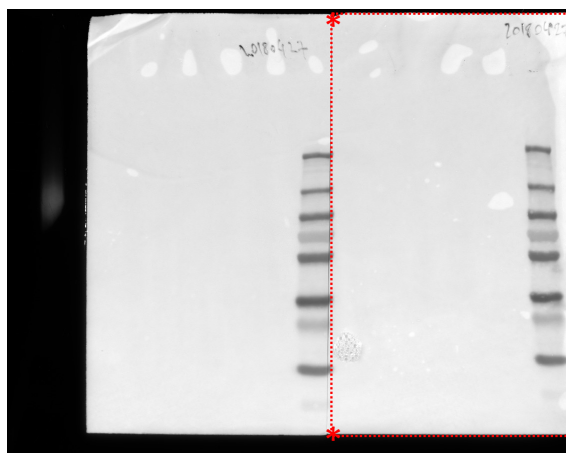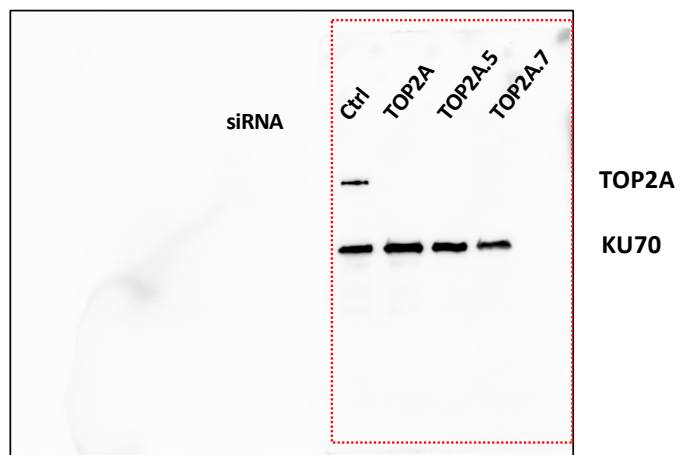

Supplement: Figure 3—figure supplement 1—source data 1. — Membrane (left panel) and hybridization signals (right panel) are shown. Raw images were acquired using the ChemiDoc system (Bio-Rad). Asterisks indicate the edges of cut membranes before hybridization. The section of the blot used for the final figure is indicated by the dashed rectangle. [file elife-65184-fig3-figsupp1-data1.pdf]

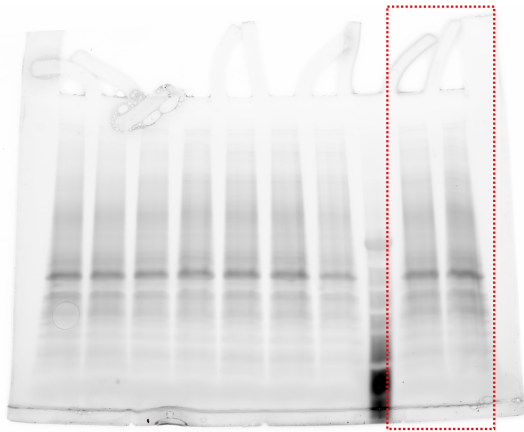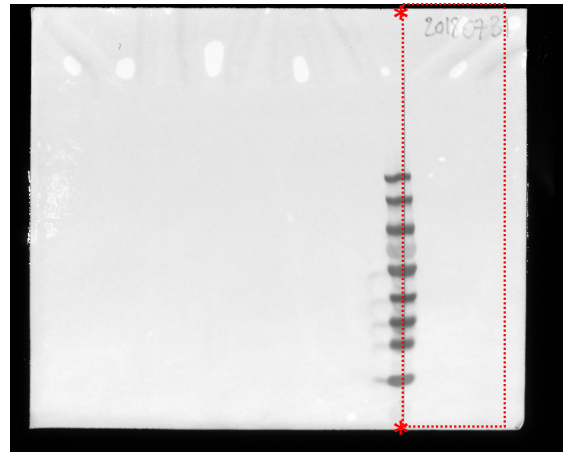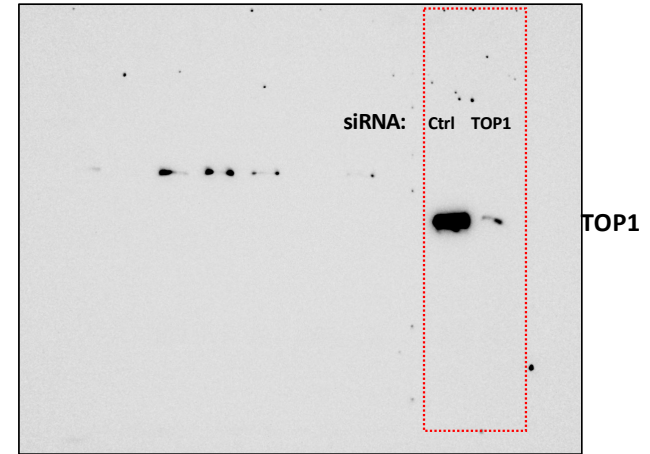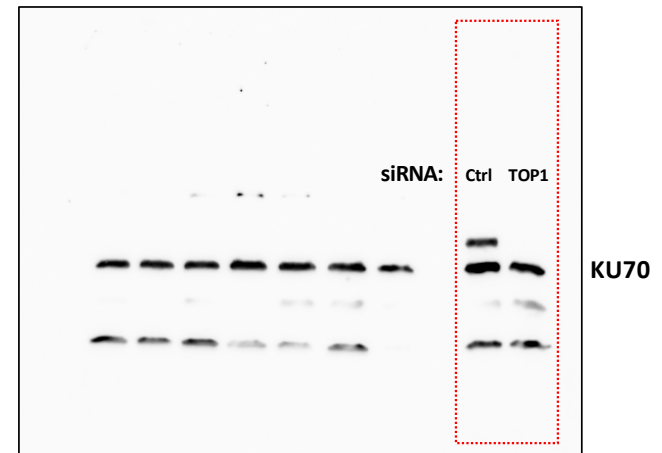

Supplement: Figure 6—source data 1. — Total proteins (stain-free signal, left panel), membrane (center panel), and hybridization signals (two right panels) are shown. Raw images were acquired using the ChemiDoc system (Bio-Rad). Asterisks indicate the edges of cut membranes before hybridization. The section of the blot used for the final figure is indicated by the dashed rectangle. [file elife-65184-fig6-data1.pdf]

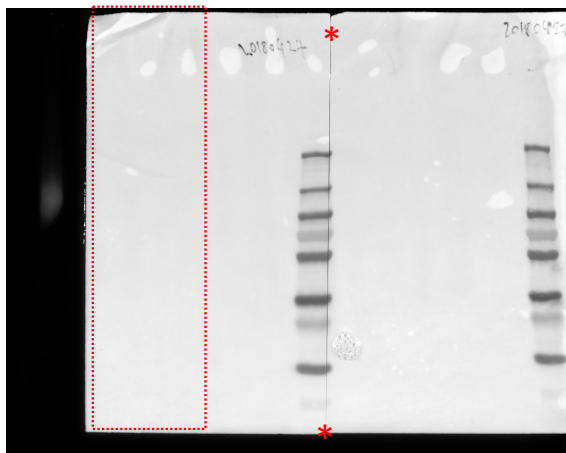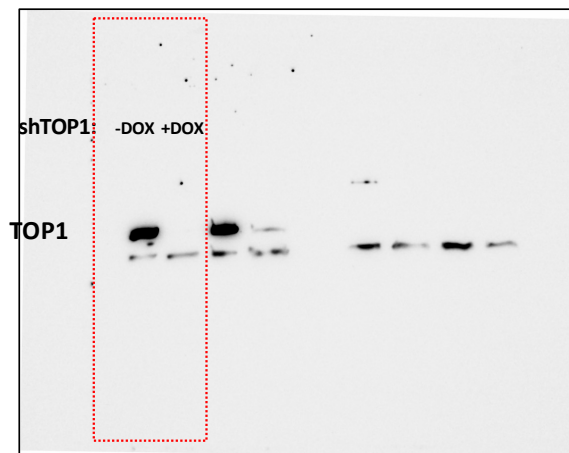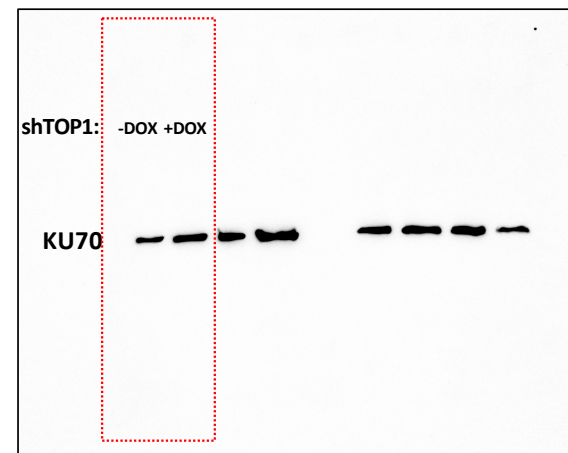

Supplement: Figure 6—source data 2. — Membrane (left panel) and hybridization signals (center and right panels) are shown. Raw images were acquired using the ChemiDoc system (Bio-Rad). Asterisks indicate the edges of cut membranes before hybridization. The section of the blot used for the final figure is indicated by the dashed rectangle. [file elife-65184-fig6-data2.pdf]

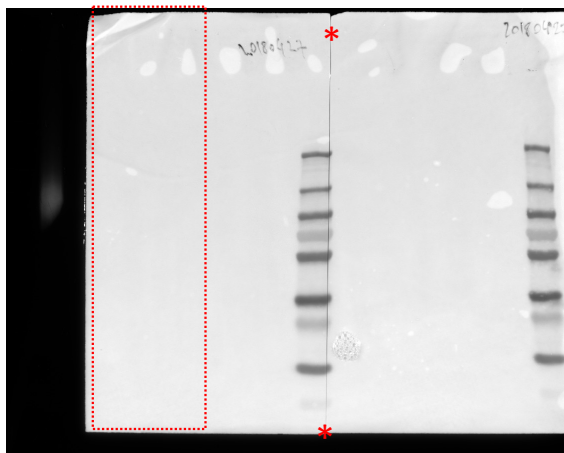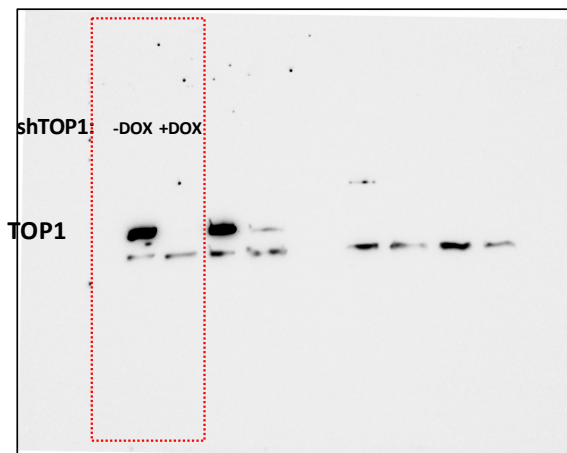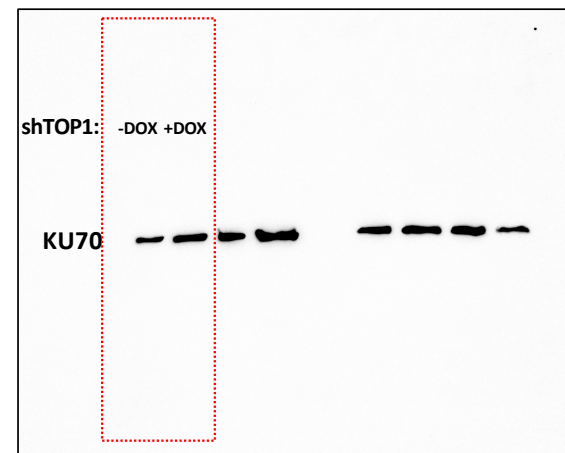

Figure 6-source data 2

Supplement: Source data 1. [file elife-65184-data1.zip › Source data Raw/Figure 6-source data 2/Figure 6-source data 2.pdf]

## Slide 1
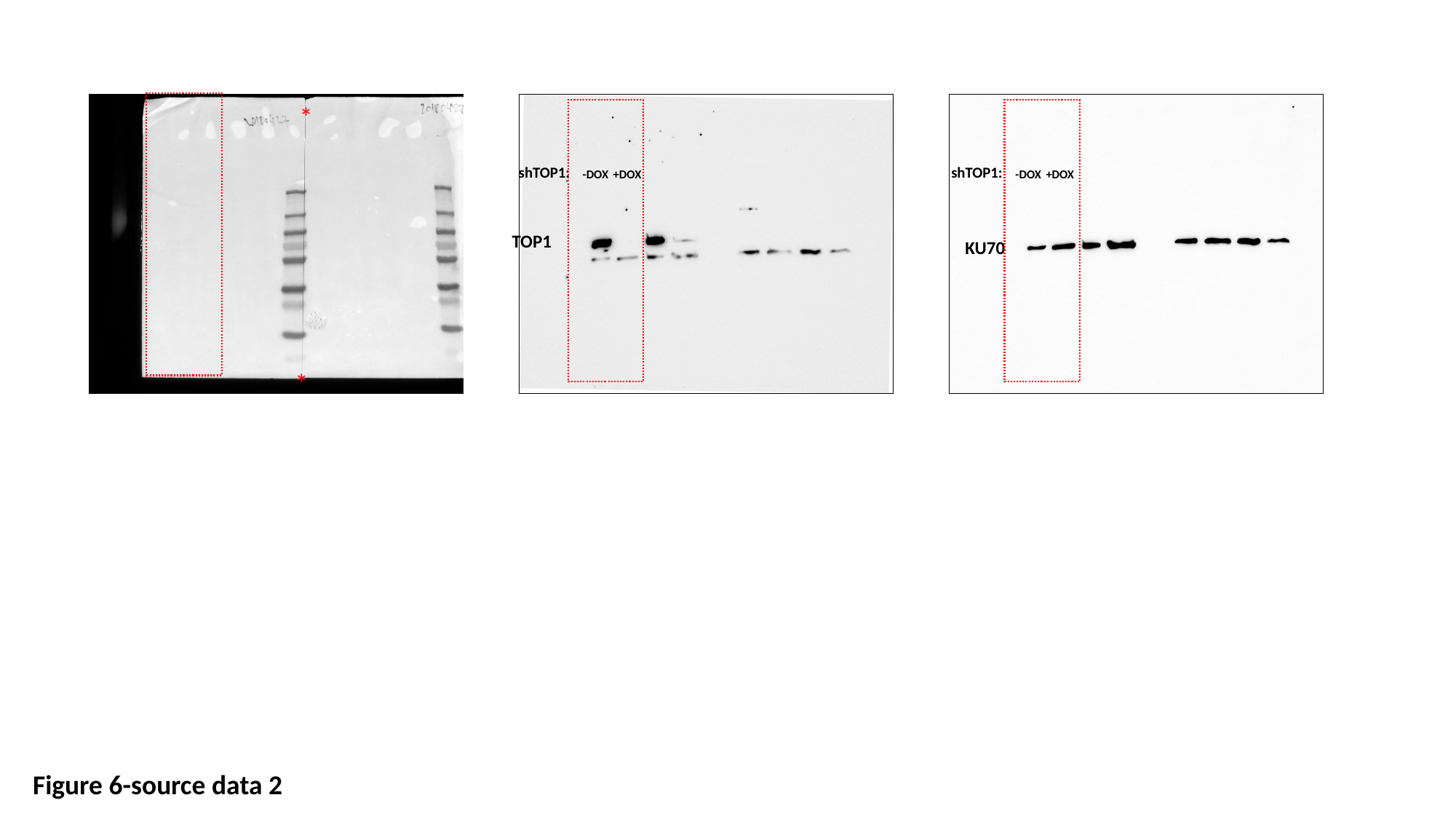

*
shTOP1:
shTOP1:
-DOX
+DOX
-DOX
+DOX
TOP1
KU70
*
Figure 6-source data 2

Supplement: Source data 1. [file elife-65184-data1.zip › Source data Raw/Figure 6-source data 2/Figure 6-source data 2.pptx]

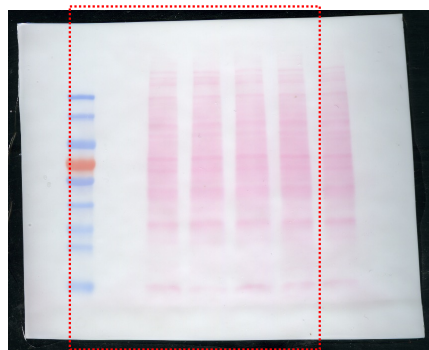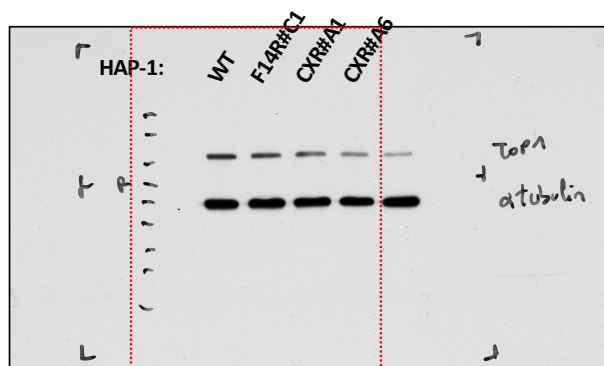

Figure 2-supplement 2-source data 1

Supplement: Source data 1. [file elife-65184-data1.zip › Source data Raw/Figure 2-supplement 2-source data 1/Figure 2-supplement 2-source data 1.pdf]

## Slide 1
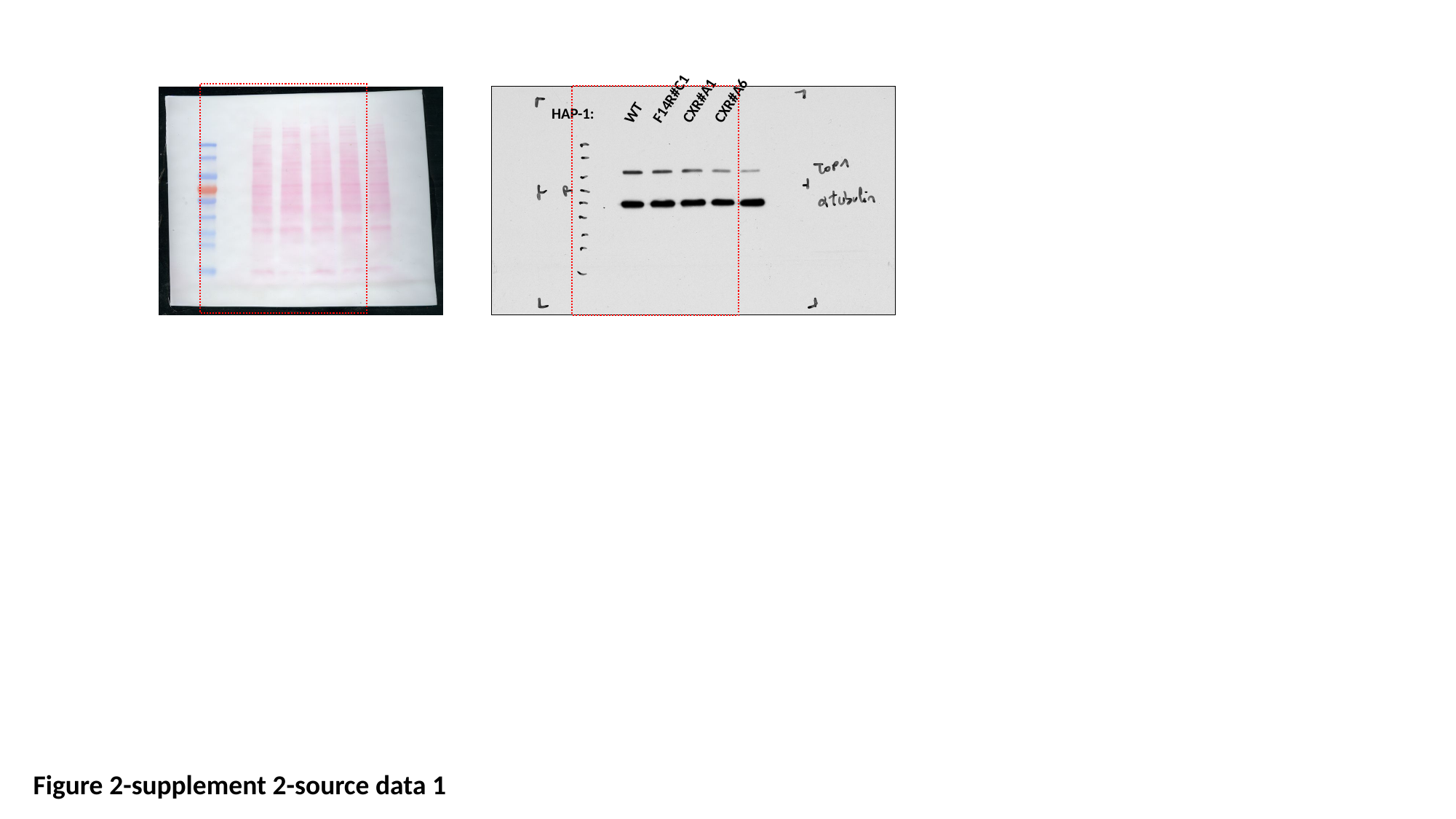

F14R#C1
CXR#A1
CXR#A6
WT
HAP-1:
Figure 2-supplement 2-source data 1

Supplement: Source data 1. [file elife-65184-data1.zip › Source data Raw/Figure 2-supplement 2-source data 1/Figure 2-supplement 2-source data 1.pptx]

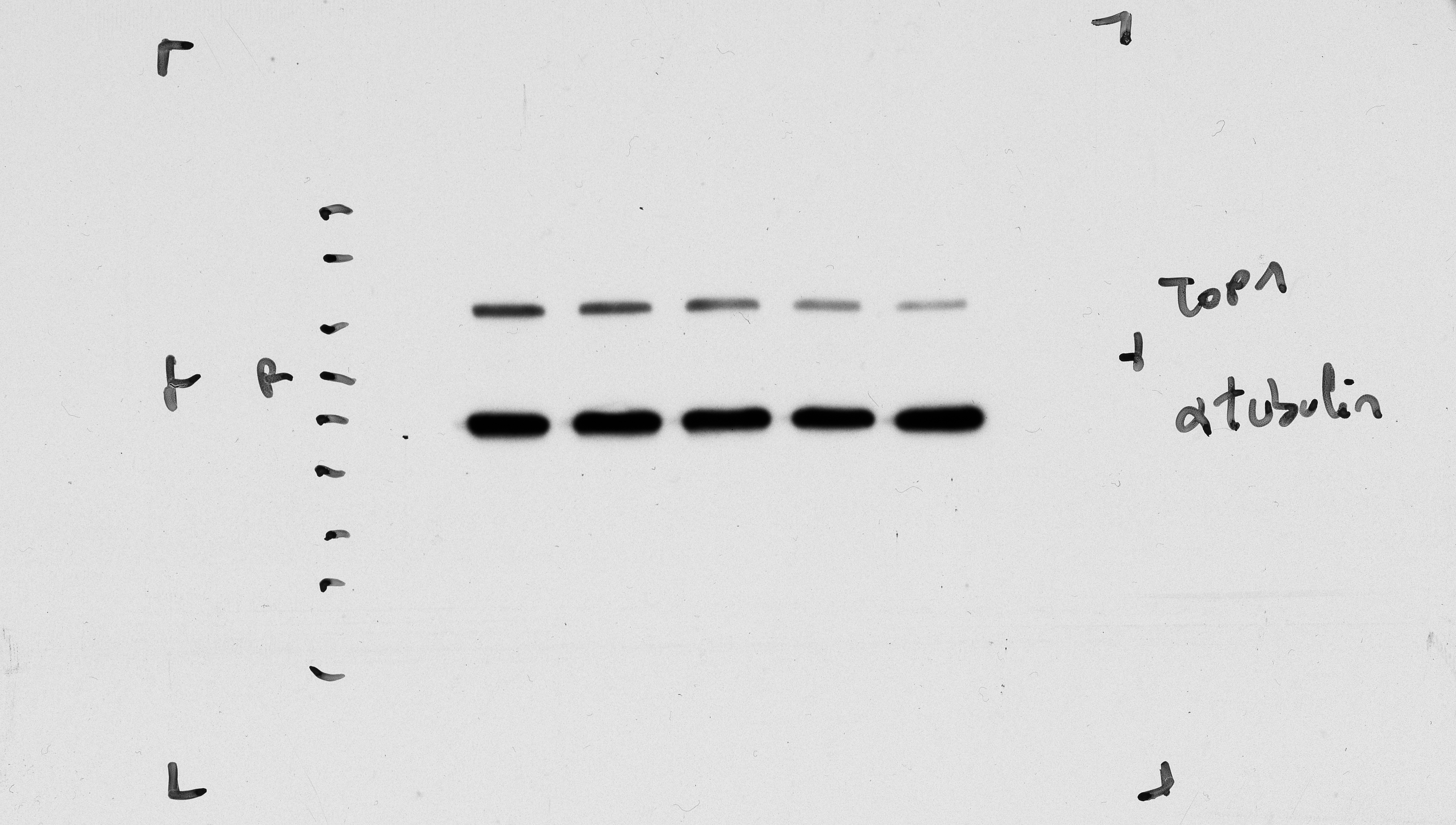

Supplement: Source data 1. [file elife-65184-data1.zip › Source data Raw/Figure 2-supplement 2-source data 1/Figure 2 -supplement 2-source data 1.tif]

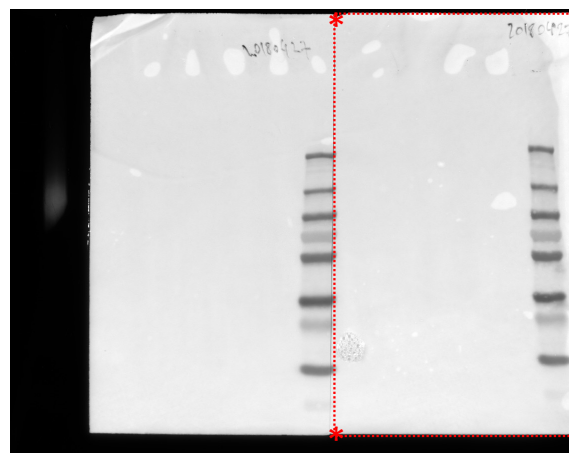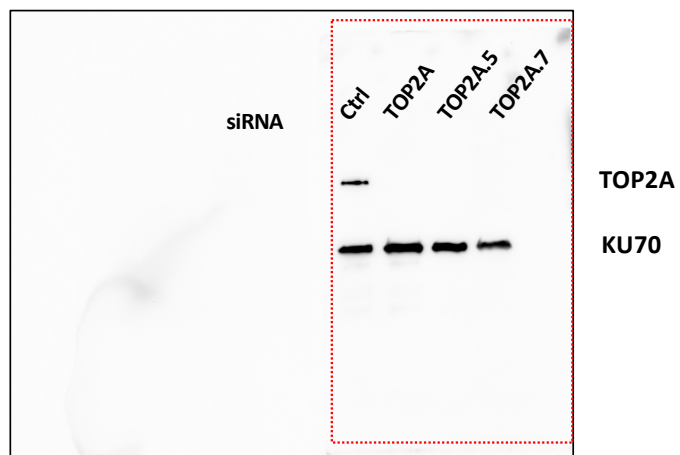

Figure 3-supplement 1-source data 1

Supplement: Source data 1. [file elife-65184-data1.zip › Source data Raw/Figure 3-supplement 1-source data 1/Figure 3-supplement 1-source data 1.pdf]

## Slide 1
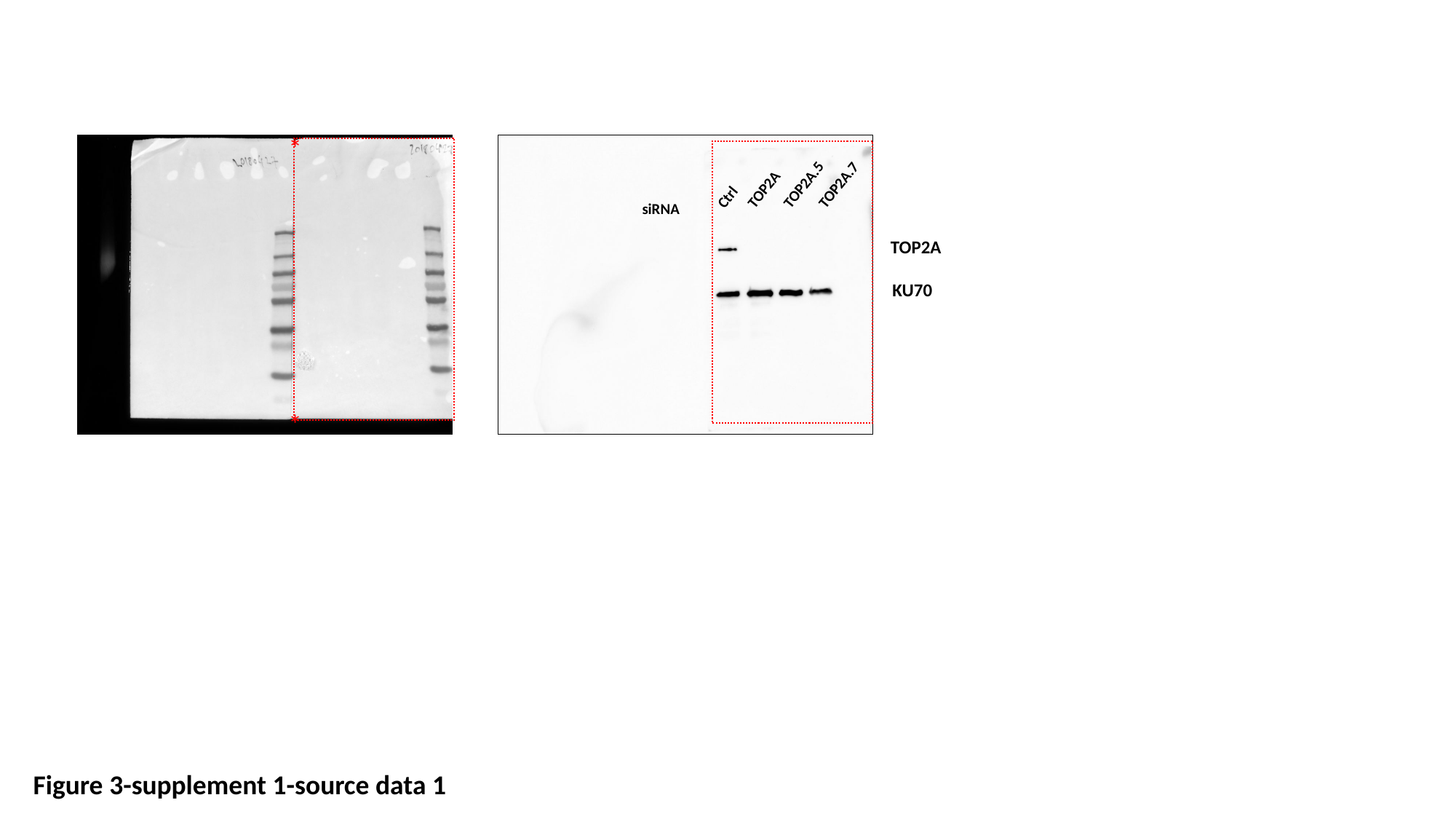

*
TOP2A.5
TOP2A.7
TOP2A
Ctrl
siRNA
TOP2A
KU70
*
Figure 3-supplement 1-source data 1

Supplement: Source data 1. [file elife-65184-data1.zip › Source data Raw/Figure 3-supplement 1-source data 1/Figure 3-supplement 1-source data 1.pptx]

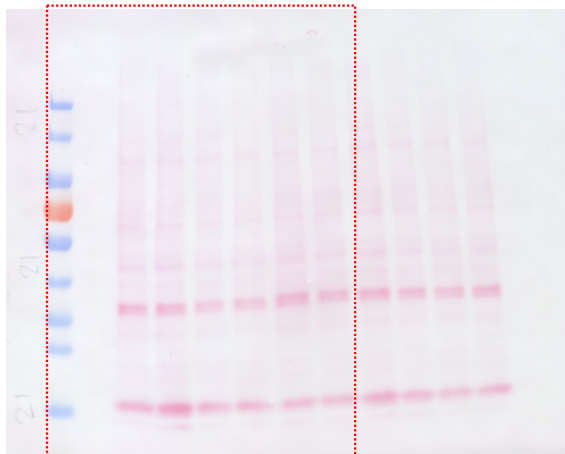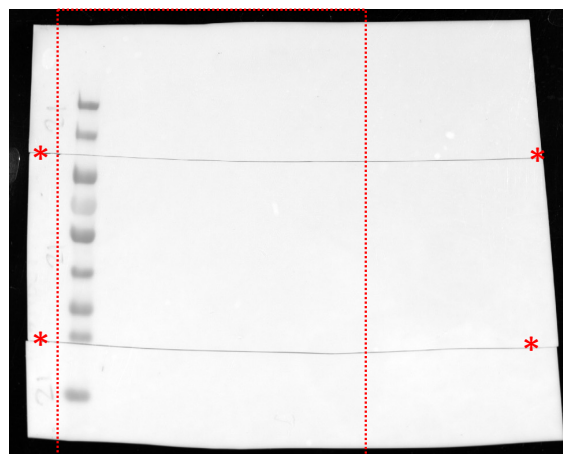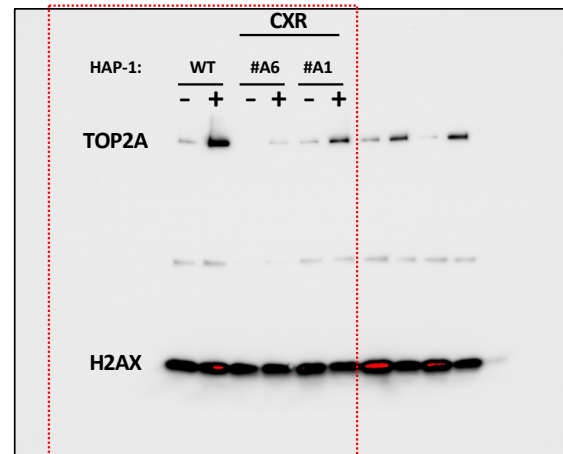

Figure 2-supplement 1-source data 1

Supplement: Source data 1. [file elife-65184-data1.zip › Source data Raw/Figure 2-suplement 1-source data 1/Figure 2-supplement 1-source data 1.pdf]

## Slide 1
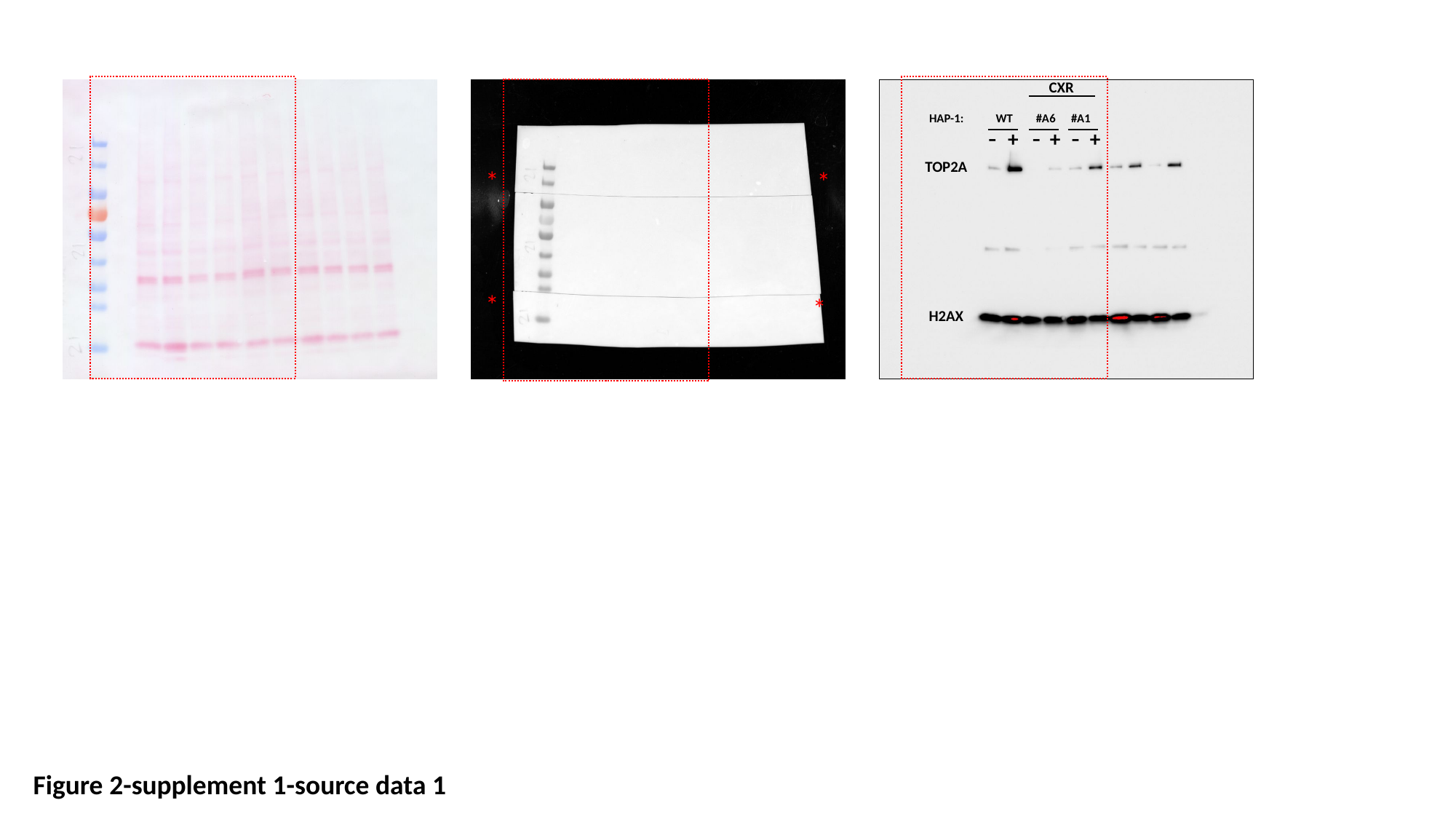

CXR
HAP-1:
WT
#A6
#A1
-
-
-
+
+
+
TOP2A
*
*
*
*
H2AX
Figure 2-supplement 1-source data 1

Supplement: Source data 1. [file elife-65184-data1.zip › Source data Raw/Figure 2-suplement 1-source data 1/Figure 2-supplement 1-source data 1.pptx]

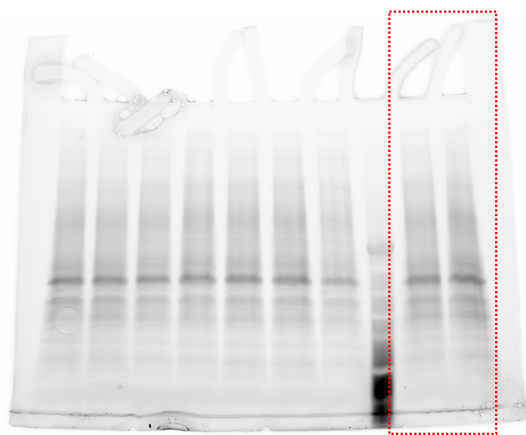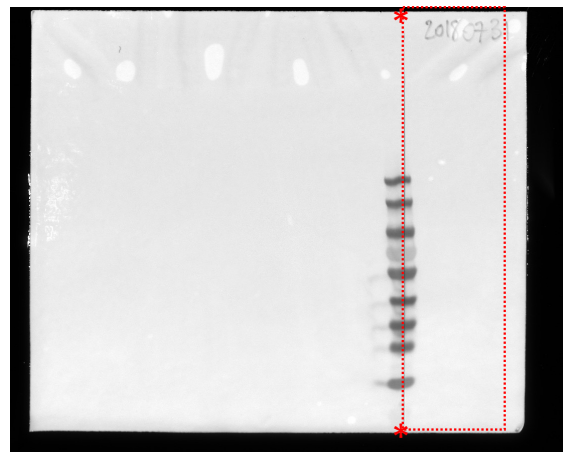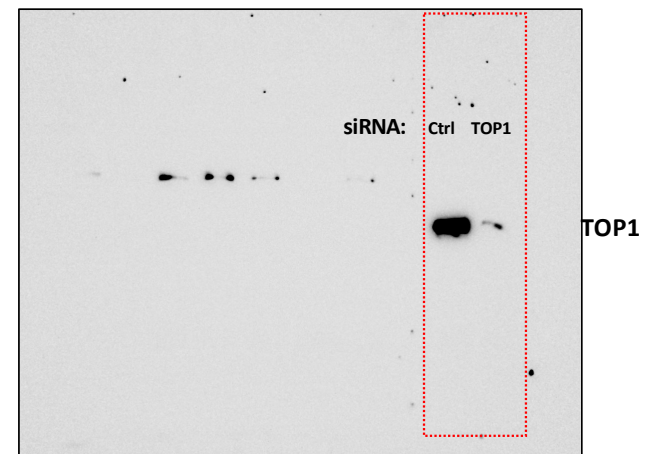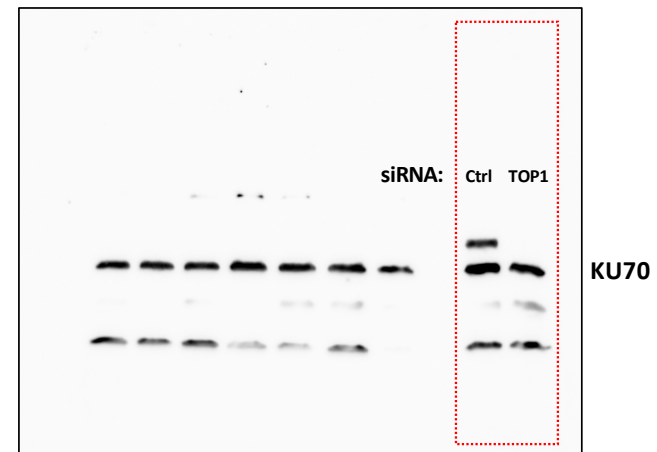

Figure 6-source data 1

Supplement: Source data 1. [file elife-65184-data1.zip › Source data Raw/Figure 6-source data 1/Figure 6-source data 1.pdf]

## Slide 1
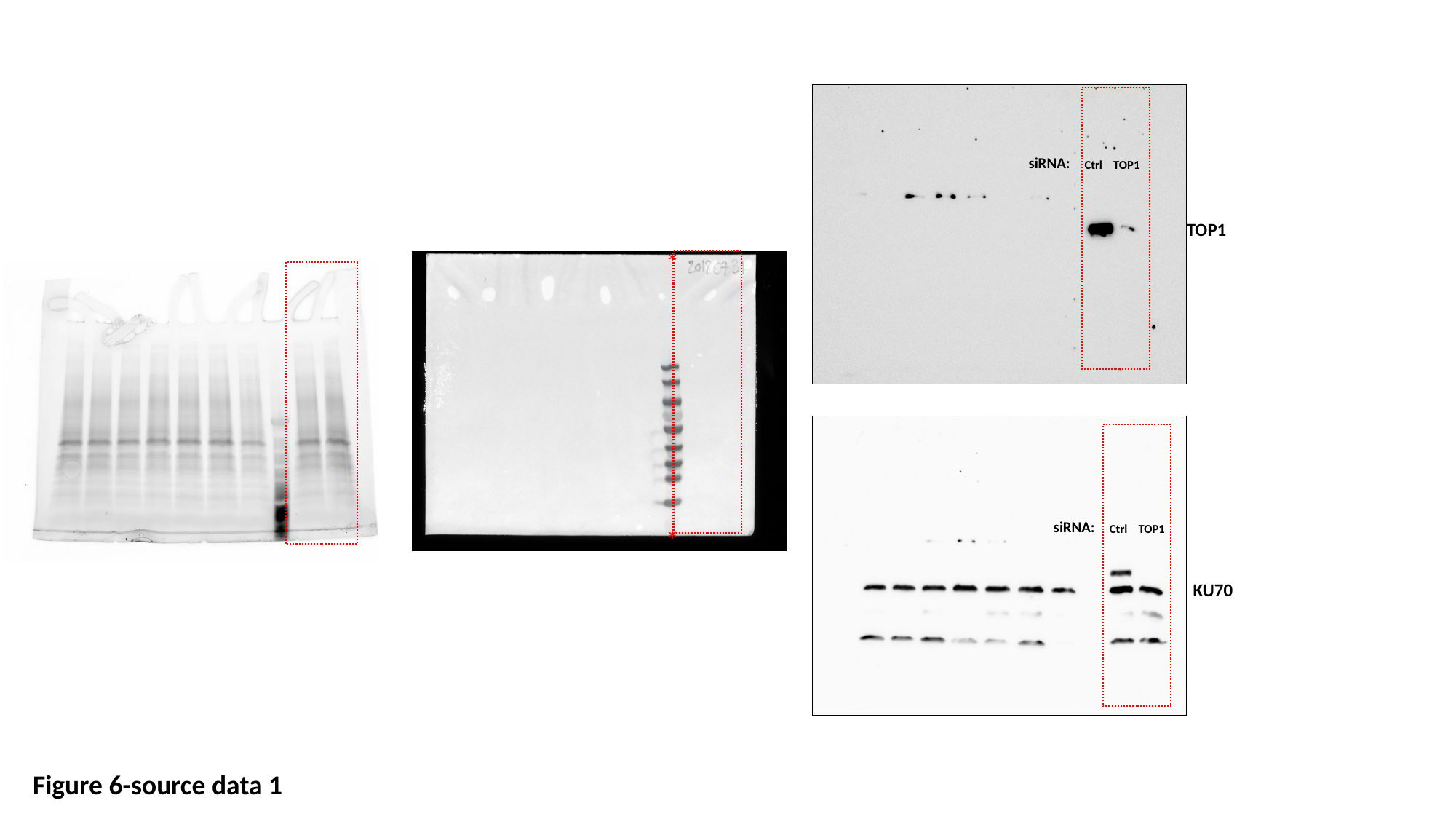

siRNA:
Ctrl
TOP1
TOP1
*
siRNA:
Ctrl
TOP1
*
KU70
Figure 6-source data 1

Supplement: Source data 1. [file elife-65184-data1.zip › Source data Raw/Figure 6-source data 1/Figure 6-source data 1.pptx]

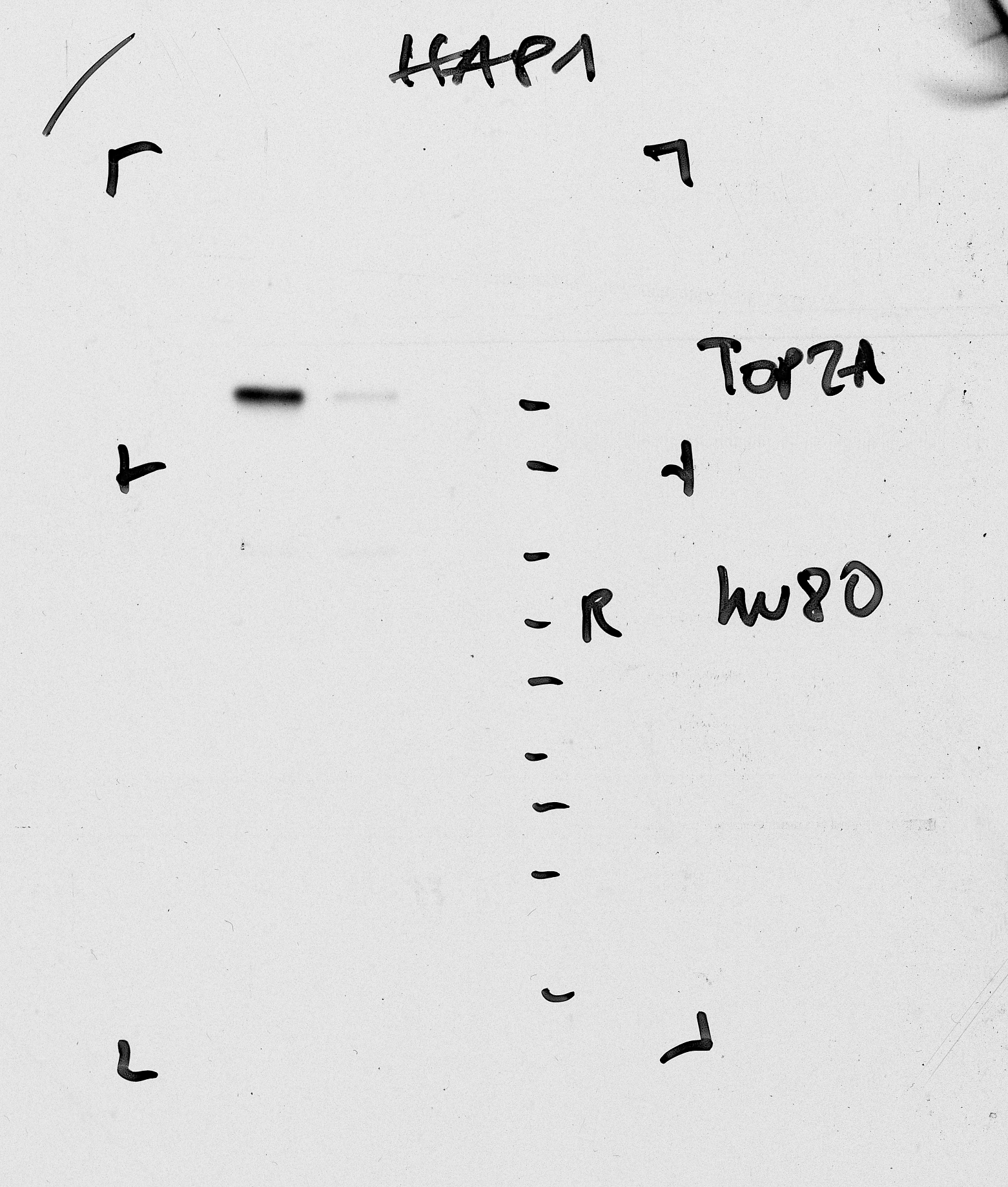

Supplement: Source data 1. [file elife-65184-data1.zip › Source data Raw/Figure 2-source data 1/Figure 2 -source data 1 (2).tif]

## Slide 1
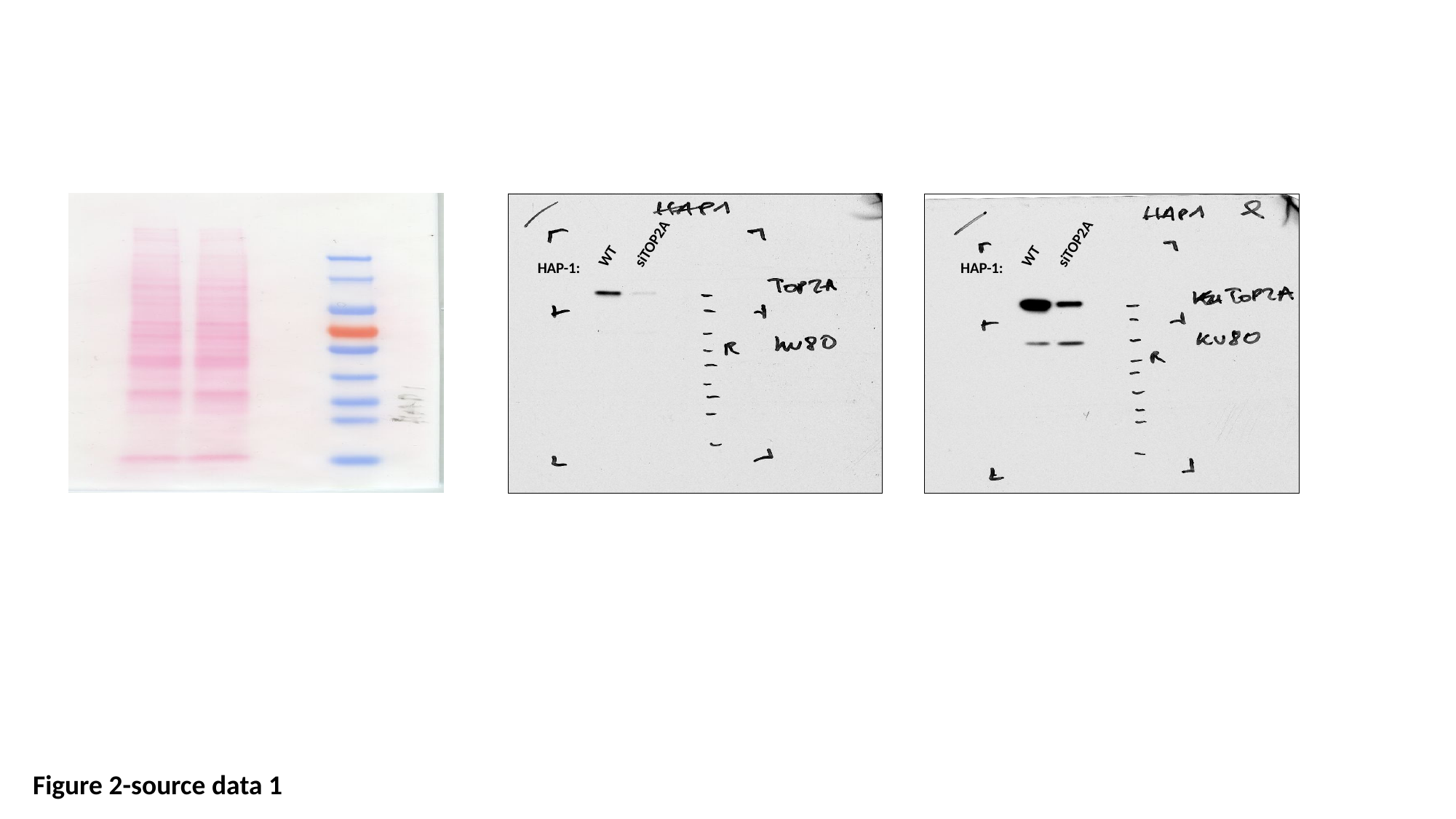

siTOP2A
siTOP2A
WT
WT
HAP-1:
HAP-1:
Figure 2-source data 1

Supplement: Source data 1. [file elife-65184-data1.zip › Source data Raw/Figure 2-source data 1/Figure 2-source data 1.pptx]

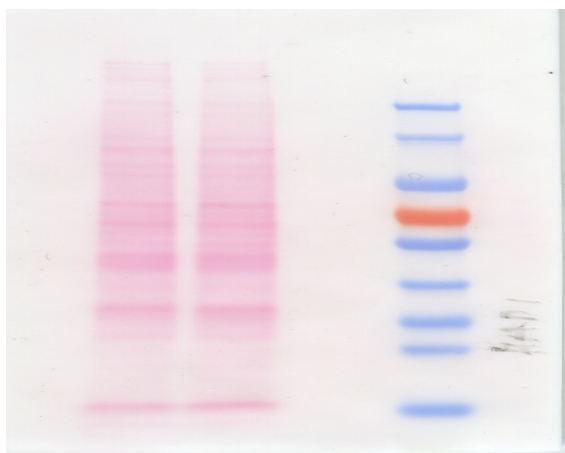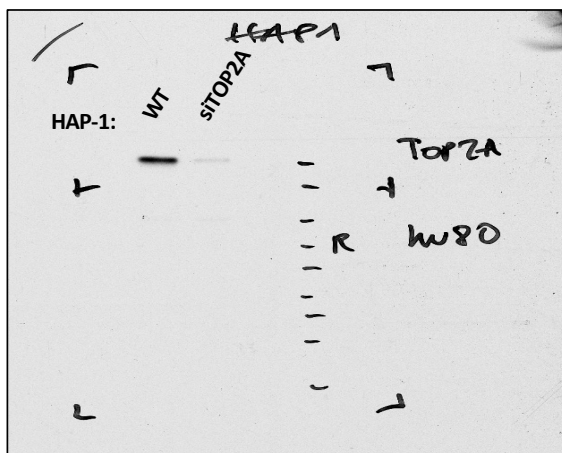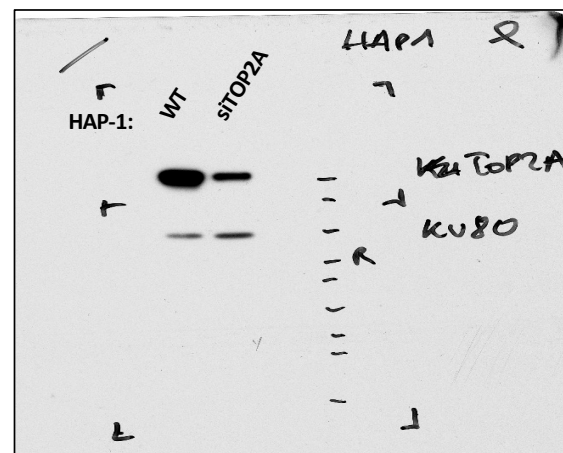

Figure 2-source data 1

Supplement: Source data 1. [file elife-65184-data1.zip › Source data Raw/Figure 2-source data 1/Figure 2-source data 1.pdf]

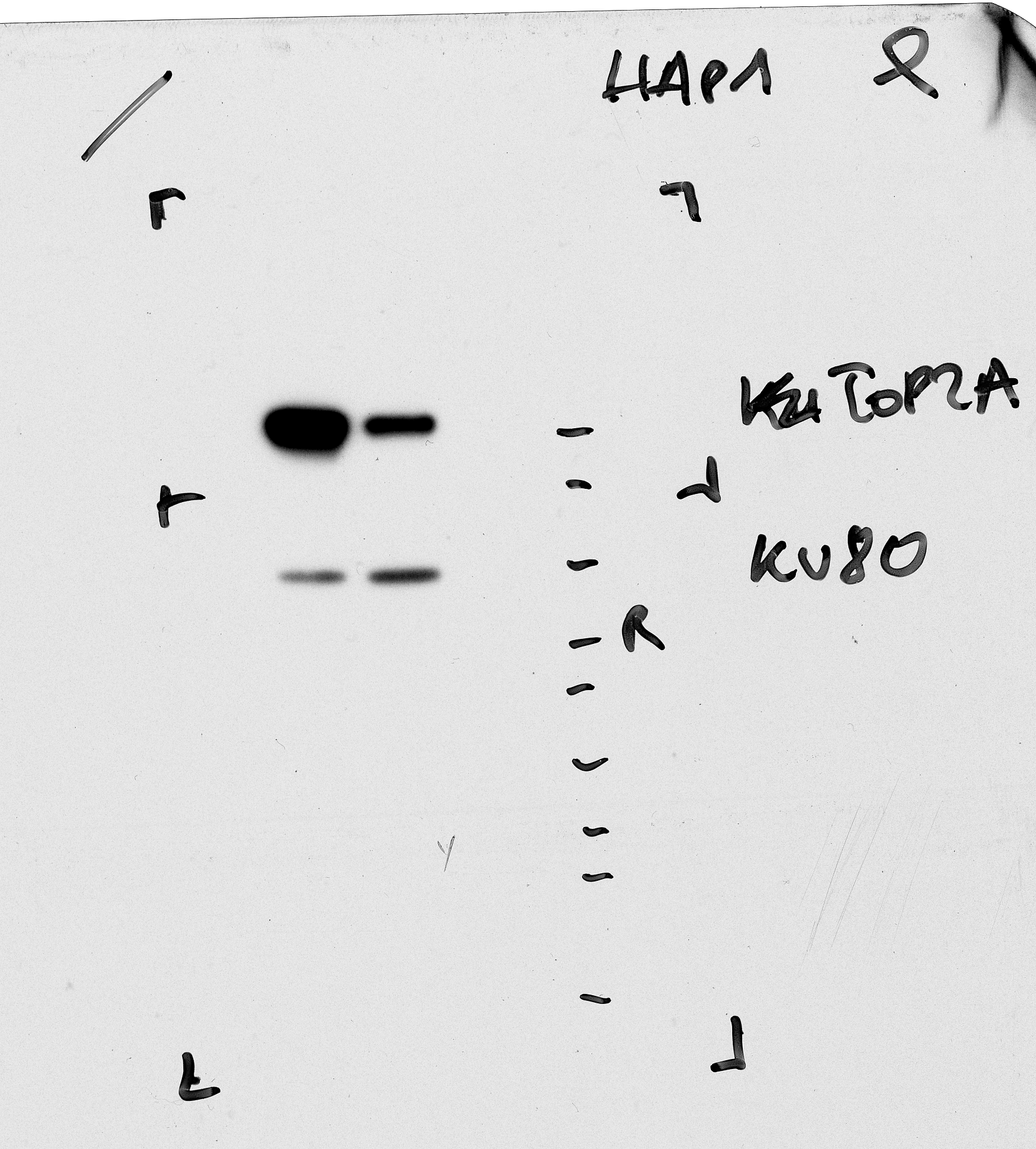

Supplement: Source data 1. [file elife-65184-data1.zip › Source data Raw/Figure 2-source data 1/Figure 2 -source data 1 (1).tif]

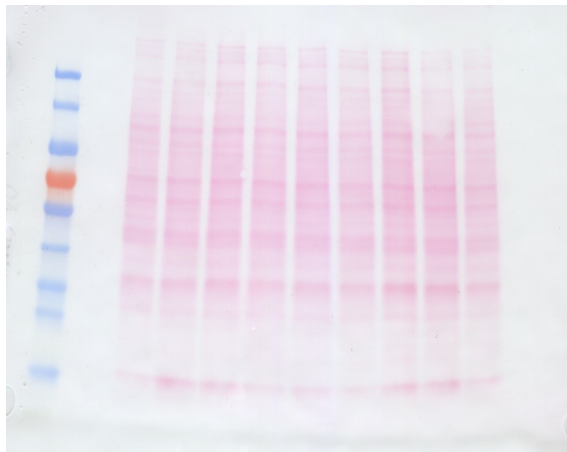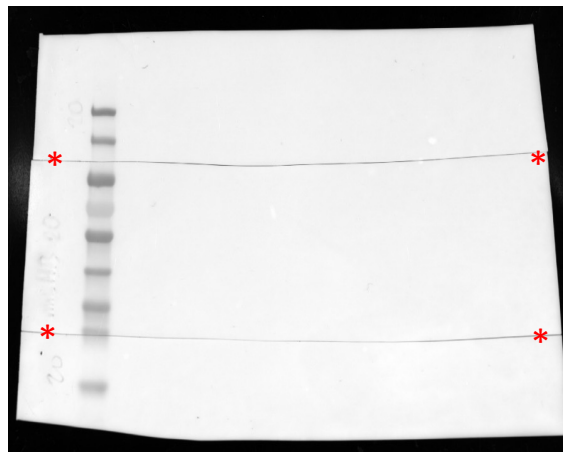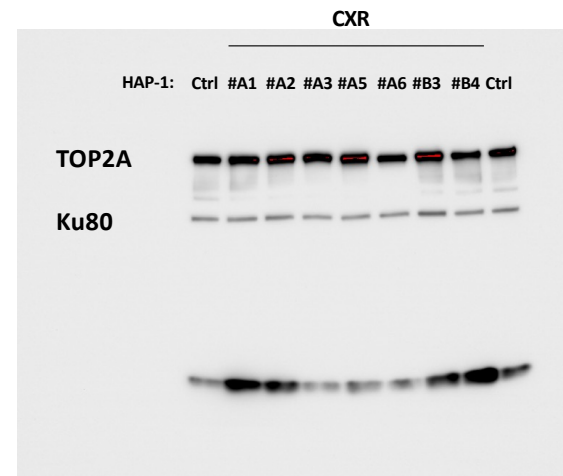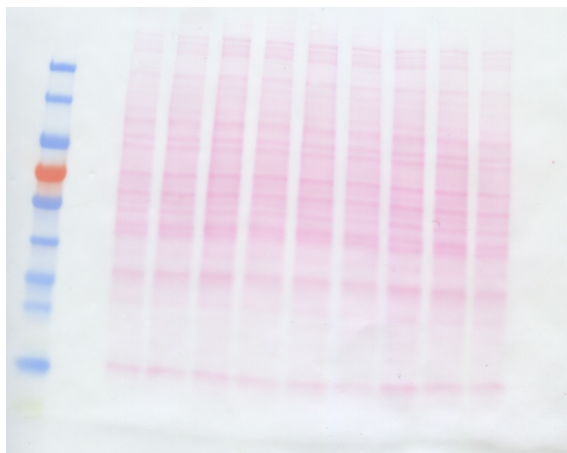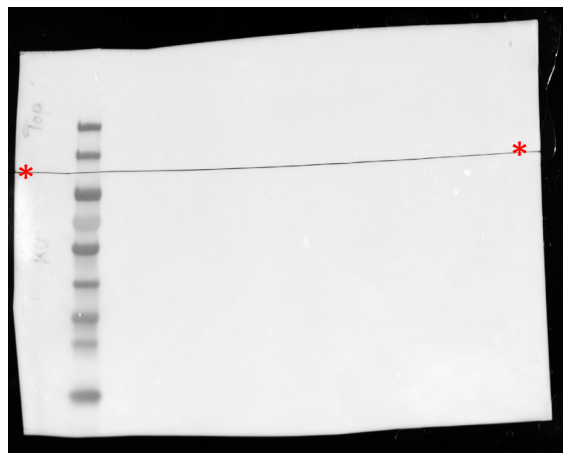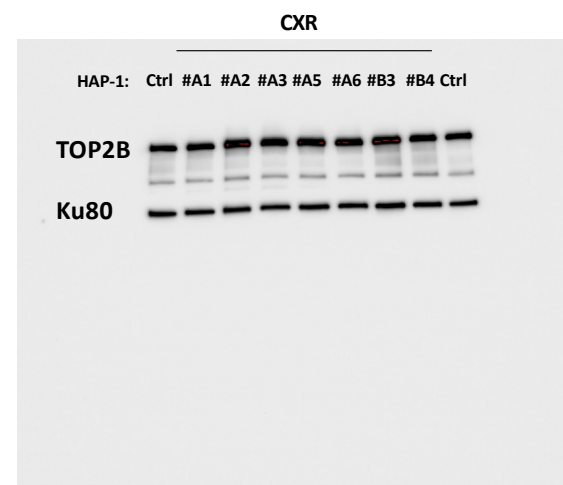

Figure 1-source data 1

Supplement: Source data 1. [file elife-65184-data1.zip › Source data Raw/Figure 1-source data 1/Figure 1-source data 1.pdf]

## Slide 1
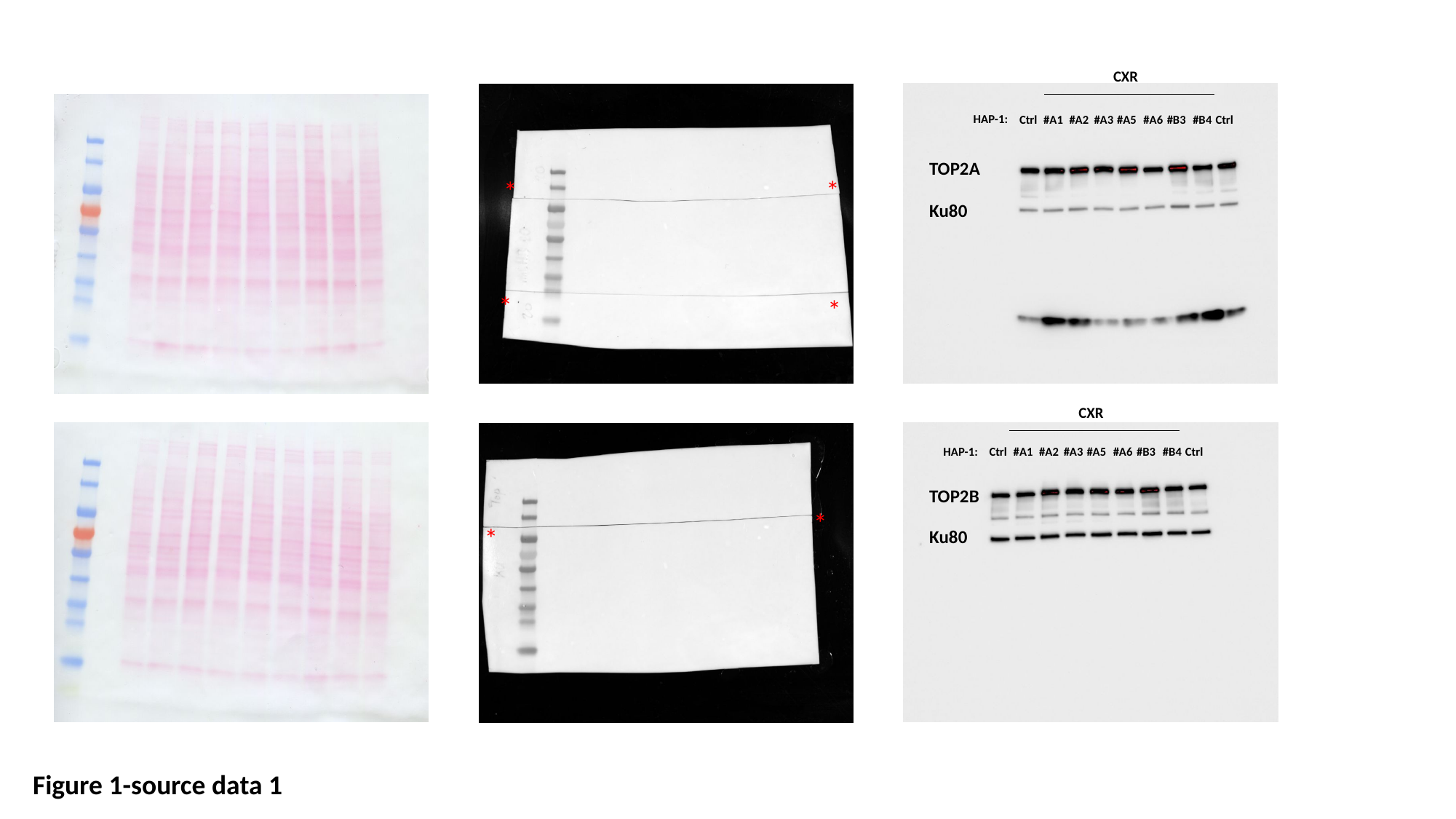

CXR
HAP-1:
Ctrl
#A1
#A2
#A3
#A5
#A6
#B3
#B4
Ctrl
TOP2A
*
*
Ku80
*
*
CXR
Ctrl
#A1
#A2
#A3
#A5
#A6
#B3
#B4
Ctrl
HAP-1:
TOP2B
*
*
Ku80
Figure 1-source data 1

Supplement: Source data 1. [file elife-65184-data1.zip › Source data Raw/Figure 1-source data 1/Figure 1-source data 1.pptx]

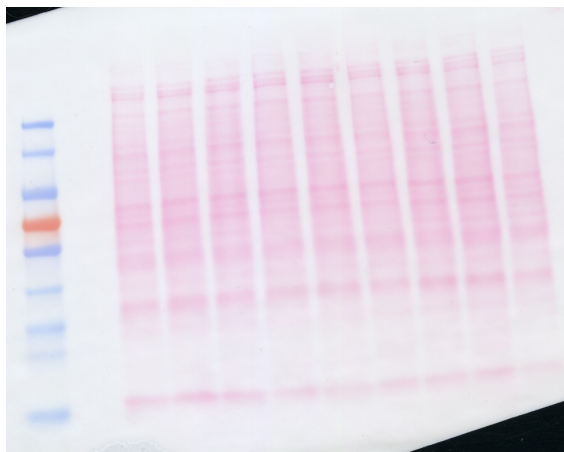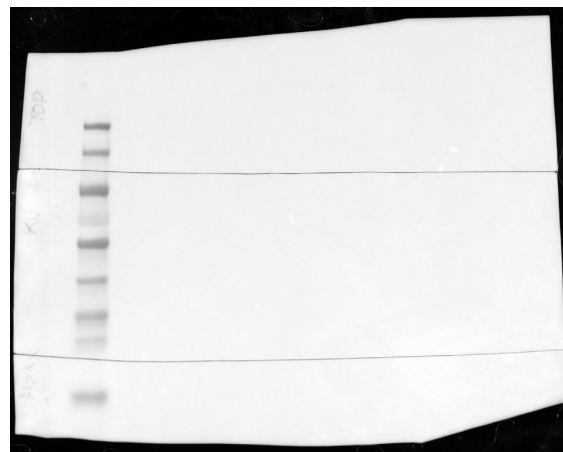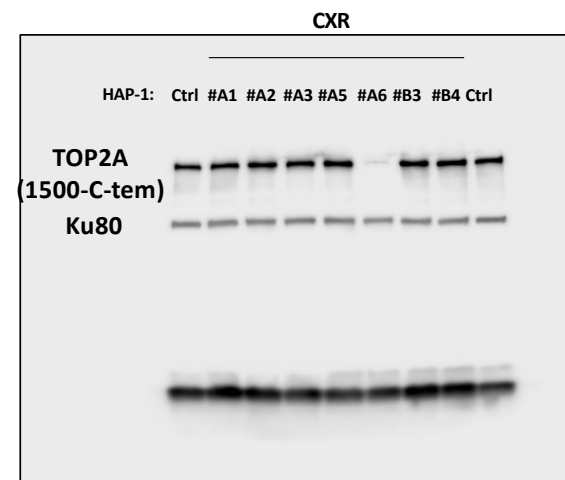

**Figure 1-supplement 1-source data 1**

Supplement: Source data 1. [file elife-65184-data1.zip › Source data Raw/Figure 1-supplement 1-source data 1/Figure 1-supplement 1-source data 1.pdf]

## Slide 1
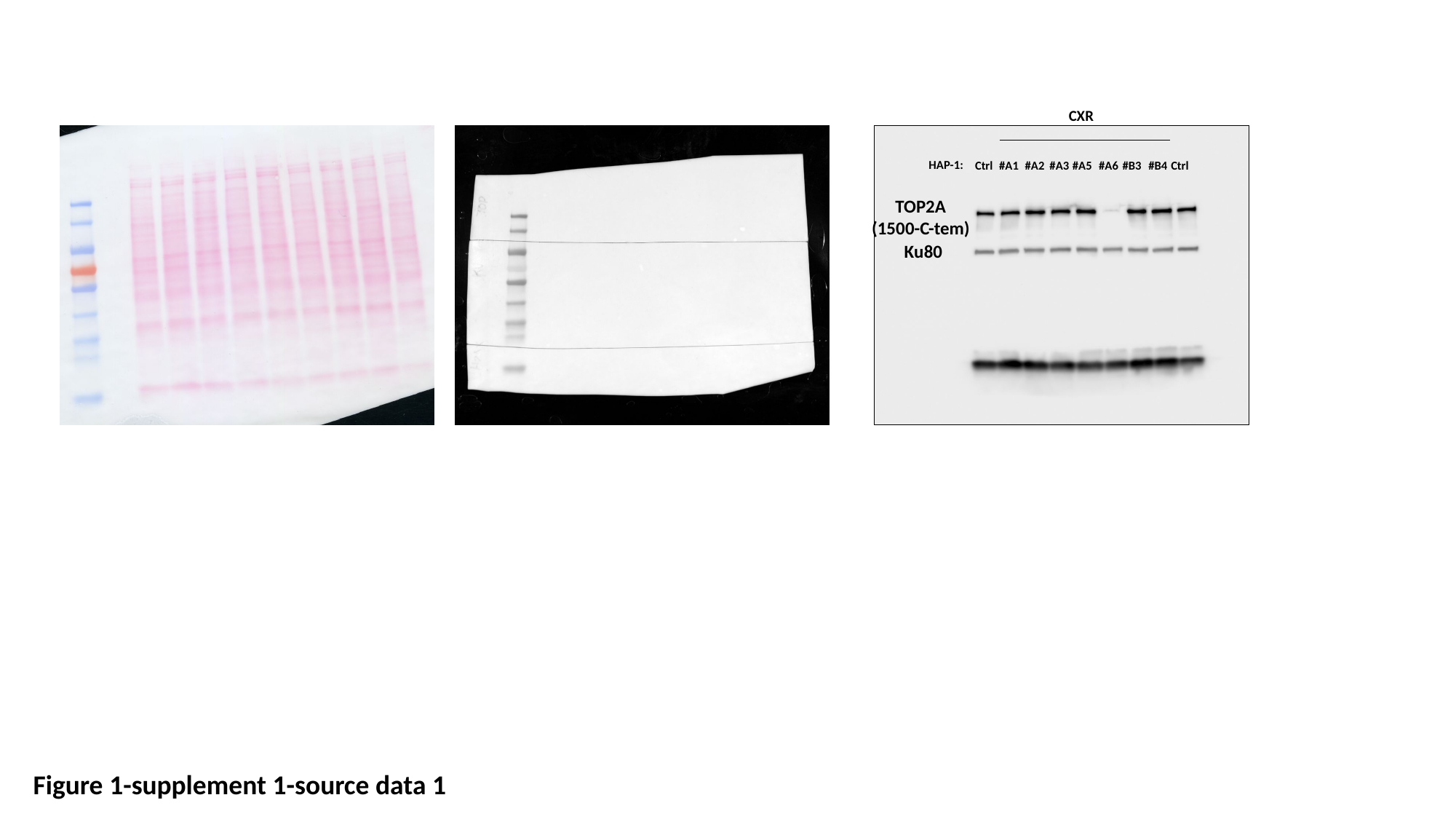

CXR
HAP-1:
Ctrl
#A1
#A2
#A3
#A5
#A6
#B3
#B4
Ctrl
TOP2A
(1500-C-tem)
Ku80
Figure 1-supplement 1-source data 1

Supplement: Source data 1. [file elife-65184-data1.zip › Source data Raw/Figure 1-supplement 1-source data 1/Figure 1-supplement 1-source data 1.pptx]
