# Supplementary material for: Transcription-associated topoisomerase 2α (TOP2A) activity is a major effector of cytotoxicity induced by G-quadruplex ligands
Source: eLife. 2021 Jun 28;10:e65184. doi: 10.7554/eLife.65184 (PMC8279764; doi:10.7554/eLife.65184)
Supplement: Supplementary file 1. — Topoisomerase 2α (TOP2A) mutation present in CXR and F14R clones are indicated in the first column. [file elife-65184-supp1.docx]

| ***TOP2A* mutation** |  | **CX-5461** | | **PDS** | | **Etoposide** | | | **F14512** | | | **Nocodazole** | | |
| --- | --- | --- | --- | --- | --- | --- | --- | --- | --- | --- | --- | --- | --- | --- |
|  |  | **IC_50_ value (µM)** | **RI*** | **IC_50_ value (µM)** | **RI*** | **IC_50_ value (µM)** | **RI*** | **IC_50_ value (nM)** | | **RI*** | **IC_50_ value (µM)** | | **RI*** |  |
|  | **HAP1 WT** | **0.03** |  | **4.20** |  | **0.05** |  | **nd** | |  | **0.02** | |  |  |
| **F85I** | **CXR #A1** | **0.29** | **8.91** | **17.09** | **4.07** | **0.39** | **8.15** | **nd** | | **nd** | **0.02** | | **1.10** |  |
| **S654N** | **CXR #A2** | **0.27** | **8.30** | **11.50** | **2.74** | **0.72** | **14.92** | **nd** | | **nd** | **0.02** | | **0.90** |  |
| **F85I** | **CXR #A3** | **0.22** | **6.76** | **14.94** | **3.56** | **0.45** | **9.35** | **nd** | | **nd** | **0.02** | | **0.90** |  |
| **F85I** | **CXR #A5** | **0.26** | **7.94** | **18.27** | **4.35** | **0.52** | **10.79** | **nd** | | **nd** | **0.02** | | **0.90** |  |
| **Splicing site** | **CXR #A6** | **0.26** | **8.00** | **15.63** | **3.72** | **0.64** | **13.42** | **nd** | | **nd** | **0.02** | | **0.90** |  |
| **L703I** | **CXR #B3** | **0.34** | **10.15** | **17.57** | **4.19** | **0.91** | **19.02** | **nd** | | **nd** | **0.02** | | **1.00** |  |
| **F85I** | **CXR #B4** | **0.26** | **7.82** | **18.41** | **4.39** | **0.51** | **10.67** | **nd** | | **nd** | **0.02** | | **0.90** |  |
|  | **HAP1 WT** | **0.04** |  | **4.16** |  | **0.05** |  | **2.73** | |  | **0.02** | |  |  |
| **Y590H** | **F14R #A2** | **0.33** | **9.15** | **12.94** | **3.11** | **0.27** | **5.01** | **40.83** | | **14.93** | **0.02** | | **1.23** |  |
| **Y590H** | **F14R #A3** | **0.20** | **5.64** | **10.95** | **2.64** | **0.22** | **4.11** | **28.82** | | **10.54** | **0.02** | | **1.20** |  |
| **W414L** | **F14R #A5** | **0.35** | **9.71** | **14.74** | **3.55** | **0.29** | **5.36** | **42.07** | | **15.39** | **0.02** | | **1.20** |  |
| **G777D** | **F14R #A6** | **0.13** | **3.58** | **7.76** | **1.87** | **0.42** | **7.69** | **29.95** | | **10.95** | **0.02** | | **1.32** |  |
| **P593S** | **F14R #B5** | **0.41** | **10.25** | **34.83** | **8.37** | **0.31** | **6.20** | **41.61** | | **15.24** | **0.02** | | **1.32** |  |
| **W414L** | **F14R #C1** | **0.60** | **16.82** | **17.37** | **4.18** | **0.35** | **6.38** | **42.50** | | **15.54** | **0.02** | | **1.13** |  |
| **P890L** | **F14R #C6** | **0.34** | **9.55** | **8.41** | **2.02** | **0.18** | **3.23** | **33.20** | | **12.14** | **0.02** | | **0.99** |  |

***RI: Resistance Index; ratio** $\frac{\boldsymbol{IC}_{\boldsymbol{50}} \left( \boldsymbol{CXR} \right)\boldsymbol{or (F}\boldsymbol{14}\boldsymbol{R)}}{\boldsymbol{IC}_{\boldsymbol{50}}\left( \boldsymbol{HAP}\boldsymbol{1 WT} \right)}$
