# Supplementary material for: Transcription-associated topoisomerase 2α (TOP2A) activity is a major effector of cytotoxicity induced by G-quadruplex ligands
Source: eLife. 2021 Jun 28;10:e65184. doi: 10.7554/eLife.65184 (PMC8279764; doi:10.7554/eLife.65184)
Supplement: Supplementary file 2. — Nucleotide changes and resulting amino acid modifications are indicated for each mutated gene. 1Topoisomerase 2α (TOP2A) mutations found by a manual analysis of RNA-seq data. [file elife-65184-supp2.docx]

| CXR#A1 | Mutation (nt) | Mutation (aa) | CXR#A2 | Mutation (nt) | Mutation (aa) | CXR#A3 | Mutation  (nt) | Mutation  (aa) |
| --- | --- | --- | --- | --- | --- | --- | --- | --- |
| BPTF | c.1775A>G | p.Lys592Arg | ADCK2 | c.1357G>T | p.Val453Phe | BPTF | c.1775A>G | p.Lys592Arg |
| DOK4 | c.514C>T | p.Pro172Ser | ARHGEF12 | c.4335C>G | p.Ile1445Met | DOK4 | c.514C>T | p.Pro172Ser |
| EIF2B1 | c.778G>A | p.Ala260Thr | B4GALNT4 | c.2613G>T | p.Glu871Asp | EIF2B1 | c.778G>A | p.Ala260Thr |
| KCTD5 | c.586G>T | p.Glu196* | CALR | c.301C>T | p.Gln101* | KCTD5 | c.586G>T | p.Glu196* |
| KIF13A | c.3982G>T | p.Ala1328Ser | JDP2 | c.515A>T | p.Glu172Val | KIF13A | c.3982G>T | p.Ala1328Ser |
| LATS2 | c.2732C>T | p.Pro911Leu | KIAA1211 | c.3314C>A | p.Ala1105Glu | LATS2 | c.2732C>T | p.Pro911Leu |
| RBM26 | c.749C>T | p.Thr250Ile | TOX2 | c.498C>A | p.Ser166Arg | RBM26 | c.749C>T | p.Thr250Ile |
| SPRED2 | c.1232G>T | p.Gly411Val | **TOP2A^1^** | **c.253T>A** | **p.Phe85Ile** | SPRED2 | c.1232G>T | p.Gly411Val |
| SPRED2 | c.1231G>T | p.Gly411Cys |  | | | SPRED2 | c.1231G>T | p.Gly411Cys |
| TOP2A | **c.253T>A** | **p.Phe85Ile** |  |  |  | **TOP2A** | **c.253T>A** | **p.Phe85Ile** |
| CXR#A5 |  |  | **CXR#A6** |  |  | **CXR#B3** |  |  |
| BPTF | c.1775A>G | p.Lys592Arg | G3BP1 | c.524A>G | p.Asp175Gly | ETNK1 | c.229G>T | p.Ala77Ser |
| DOK4 | c.514C>T | p.Pro172Ser | SULF2 | c.1946G>A | p.Arg649Gln | FAM219A | c.211G>A | p.Gly71Ser |
| EIF2B1 | c.778G>A | p.Ala260Thr | **TOP2A^1^** | **Splicing site** |  | GLIPR2 | c.122T>A | p.Val41Asp |
| KCTD5 | c.586G>T | p.Glu196* |  | | | KDM5B | c.1481G>T | p.Gly494Val |
| LATS2 | c.2732C>T | p.Pro911Leu |  |  |  | KDM5B | c.1480G>T | p.Gly494Cys |
| RBM26 | c.749C>T | p.Thr250Ile |  |  |  | RAF1 | c.976G>T | p.Glu326* |
| SPRED2 | c.1232G>T | p.Gly411Val |  |  |  | TBC1D24 | c.637C>A | p.Gln213Lys |
| SPRED2 | c.1231G>T | p.Gly411Cys |  |  |  | TESMIN | c.596C>A | p.Ser199Tyr |
| TOP2A | **c.253T>A** | **p.Phe85Ile** |  |  |  | **TOP2A** | **c.2107C>A** | **p.Leu703Ile** |
| CXR#B4 |  |  |  | | | | | |
| BPTF | c.1775A>G | p.Lys592Arg |  |  |  |  |  |  |
| DOK4 | c.514C>T | p.Pro172Ser |  |  |  |  |  |  |
| EIF2B1 | c.778G>A | p.Ala260Thr |  |  |  |  |  |  |
| KCTD5 | c.586G>T | p.Glu196* |  |  |  |  |  |  |
| LATS2 | c.2732C>T | p.Pro911Leu |  |  |  |  |  |  |
| RBM26 | c.749C>T | p.Thr250Ile |  |  |  |  |  |  |
| RREB1 | c.2567G>T | p.Cys856Phe |  |  |  |  |  |  |
| SPRED2 | c.1232G>T | p.Gly411Val |  |  |  |  |  |  |
| SPRED2 | c.1231G>T | p.Gly411Cys |  |  |  |  |  |  |
| TOP2A | **c.253T>A** | **p.Phe85Ile** |  |  |  |  |  |  |
